# Comparison of SNP-based and gene-based association studies in detecting rare variants using unrelated individuals

**DOI:** 10.1186/1753-6561-5-S9-S41

**Published:** 2011-11-29

**Authors:** Liping Tong, Bamidele Tayo, Jie Yang, Richard S Cooper

**Affiliations:** 1 Department of Mathematics and Statistics, Loyola University, Chicago, IL 60626, USA; 2 Department of Preventive Medicine and Epidemiology, Loyola University Medical School, Maywood, IL 60153, USA; 3 Department of Mathematics, Statistics, and Computer Science, University of Illinois at Chicago, Chicago, IL 60607, USA

## Abstract

We compare the SNP-based and gene-based association studies using 697 unrelated individuals. The Benjamini-Hochberg procedure was applied to control the false discovery rate for all the multiple comparisons. We use a linear model for the single-nucleotide polymorphism (SNP) based association study. For the gene-based study, we consider three methods. The first one is based on a linear model, the second is similarity based, and the third is a new two-step procedure. The results of power using a subset of SNPs show that the SNP-based association test is more powerful than the gene-based ones. However, in some situations, a gene-based study is able to detect the associated variants that were neglected in a SNP-based study. Finally, we apply these methods to a replicate of the quantitative trait Q1 and the binary trait D (D = 1, affected; D = 0, unaffected) for a genome-wide gene search.

## Background

Our aims are (1) to compare single-nucleotide polymorphism (SNP) based and gene-based association studies and (2) to apply both methods to a genome-wide search for associated genes. To check the effect of covariates, we use linear models for the numerical traits Q1, Q2, and Q4 and a logistic model for the binary trait D. We modify the original trait value by adjusting for significant covariate effects. Then in the SNP-based association study, we delete alias SNPs and use a linear or logistic model to find the *p*-value for each SNP.

In the gene-based association study, we consider three methods. The first two methods are two major types of multilocus association study methods, and the third method is a new method that we propose. Specifically, the first method is based on multilinear regression [1]. The second method is similarity based and is useful for binary traits only [2]. The *p*-values are calculated using the cumulant-based estimation procedure [3]. These two multilocus association study methods can have reduced power as a result of increased degrees of freedom [4]. To solve this problem, we propose a two-step procedure that first classifies and collapses multilocus genotypes and then uses the classic *T* and Pearson chi-square statistics to perform an association test. To reduce computational time, the overall *p*-value is obtained using a self-adjusted permutation procedure, as described by Knuth [5].

From our analysis, we conclude that the detection of association signals for rare variants is a challenging problem. The SNP-based association study is more powerful in general. However, the gene-based study can pick up some association signals neglected by a SNP-based study.

## Methods

### Data

We use the genotype data from 697 unrelated individuals in the Genetic Analysis Workshop 17 (GAW17) data set. For the first aim (comparing SNP-based and gene-based association studies), we consider all four traits and use all 200 replicates. For computational convenience, we include only a subset of genes, which is formed by the 9 true associated genes (39 SNPs) for trait Q1, the 13 true associated genes (72 SNPs) for trait Q2, the additional 15 genes (51 SNPs) for the binary trait D, and 50 other randomly selected nonassociated genes (731 SNPs). The total number of genes is 86, and they contain 893 SNPs. For the second aim (applying both methods to a genome-wide search for associated genes), we use the complete list of all the genes and SNPs, which includes 3,205 genes (24,487 SNPs). Because Q1 has the most significant association and the binary trait D has the weakest association, we consider only a fixed replicate data set for these two traits as representation.

### Effect of covariates

We considered the three covariates Sex, Age, and Smoking before conducting association studies. We use linear models for the quantitative traits Q1, Q2, and Q4 and a logistic model for the binary trait D. We also check assumptions in the models to make sure they are appropriate. For each trait and replicates, we first fit the corresponding models that include all three covariates to test whether they are significant. Then we include only the significant covariates in the model and calculate residuals. These residuals are used as new trait values to perform association studies.

### SNP-based association study

Because there are numerous rare SNPs, it is likely that SNPs at two different loci will be in complete linkage disequilibrium. That is, *R*^2^ = *D*′ = 1. We call these alias SNPs; they do not contribute additional information to an association study. Therefore, to increase power, we use only one copy of the alias SNPs. The new data set generated here is called the cleaned data. In the subset of SNPs for our first aim (comparing SNP-based and gene-based association studies), the cleaned data include 83 genes and 773 SNPs. In the complete data set for our second aim (applying both methods in a genome-wide search for associated genes), the cleaned data include 2,987 genes and 15,076 SNPs. Note that the cleaned data here are used to control the false discovery rate (FDR) only.

We assume an additive genetic model. For each trait and SNP in the cleaned data, we perform simple linear (or logistic) regression. The *p*-value to test the null hypothesis that the slope is 0 versus the alternative that the slope is not 0 is recorded. To adjust for multiple testing, we use the Benjamini-Hochberg (BH) procedure to control FDR [6]. That is, let *m* be the number of SNPs in the cleaned data, and let *P*_(_*_i_*_)_ be the *i*th ordered *p*-value. Then for a prespecified 0 <*α* < 1, define:(1)

as the BH threshold. When the tests are independent, this procedure guarantees that FDR ≤ *α*.

### Gene-based association study

In the gene-based association study, we consider all 86 genes and 893 SNPs. Likewise, we use the BH procedure to control FDR. For each gene, the multilocus genotype is defined as the composition of genotypes at all SNPs included in the gene. If the gene contains *L* SNPs, then in theory the total number of possible genotypes for the gene is 3*^L^*. However, because of rare variants and linkage disequilibrium in nearby SNPs, the actual observed number of multilocus genotypes is much less.

The first gene-based association study method is referred as GL, which is based on a linear (or logistic) model. That is, traits are regressed on all the SNPs included in the gene. The *p*-value to test the null hypothesis that all coefficients are 0 versus the alternative that at least one coefficient is not 0 is recorded.

The second gene-based method is referred as GS, which is similarity based and is used for the binary trait D only. The test statistic *D_s_* is defined as:(2)

where  is a vector of the estimated frequencies of multilocus genotypes in the affected group,  is a vector of those in the unaffected group, and *A* = (*a_ij_*) is the genotype similarity matrix [4]. Here the similarity score *a_ij_* between the *i*th and *j*th distinct genotypes is defined to be 1 minus the weighted average of absolute differences between numbers of minor alleles at each SNP of a gene. The weight is the inverse of the minor allele frequency (MAF), which accounts for the assumption that genomes that share minor alleles are more similar than those that share common ones [7]. According to Tong et al. [3], the distribution of *D_s_* under the null hypothesis can be approximated by a chi-square distribution or the difference between two chi-square distributions.

The third gene-based method is referred as G2, which has two steps. In the first step, we define a null multilocus genotype to be the one with genotype *g* = 0 at all the SNP loci, where *g* is the number of minor alleles. We then compare each distinct multilocus genotype with the null genotype and classify it into one of the following three groups: (1) *C* = 0 (not distinguishable from the null genotype); (2) *C* = −1 (negatively contributed to the trait); and (3) *C* = 1 (positively contributed to the trait). In the second step, we compare the means of the trait values for the three groups. For each gene, we estimate two *p*-values (one for the *C* = 1 group compared with the *C* = 0 group and one for the *C* = −1 group compared with the *C* = 0 group) using a permutation procedure.

In a permutation, we first randomly assign an observed trait value to each individual and then follow the two-step procedure to find the mean differences for comparison with the observed ones. To improve the efficiency of computation, we use a self-adjusted procedure to decide the number of permutations needed [5]. Specifically, we require that the standard deviation of the estimated *p*-value, denoted , be at most 10% of it. That is, let *R* be the number of permutations, and let *S* be the number of times that the mean difference in a permutation is greater than or equal to the observed mean difference. Then . The permutation stops when , which is equivalent to *S* ≥ 100*R*/(100 + *R*). The minimum number of permutations is set at 100.

### Comparison of association studies

Because the number of hypotheses in a SNP-based study is different from the number of hypotheses in a gene-based study, we have to redefine the false positive rate (FPR), the FDR, and the power for a SNP-based study to make them comparable with the gene-based values. Specifically, consider all the SNPs in a gene. If at least one minor allele contributes significantly to the trait at a predefined significance level, then this gene is detected. Then for each replicate data set, the list of significant genes is determined. In this list of genes, some are true associated variants and some are not. The observed FPR is calculated as the number of reported false positives divided by the total number of true null genotypes. The observed FDR is the number of reported false positives divided by the total number of reported positives. The power (or alternatively, true discovery rate) is the number of reported true positives divided by the total number of true alternatives. For the gene-based studies, the list of significant genes is determined directly given a predefined significance level. Likewise, the observed FPR, FDR, and power are calculated. The observed FDR versus power receiver operating characteristic (ROC) plot is presented to compare the overall performance of these methods.

## Results and discussion

### Effect of covariates

We estimate the parameter values in linear models separately for each of the 200 replicate data sets. The linear or logistic model assumptions seem appropriate. The effects of covariates are consistent in the 200 replicates. For trait Q1, Age and Smoking are significant, which explain 15% of the variation. The value of Q1 increases with age and is higher in smokers. No covariates contribute to trait Q2. For trait Q4, all three covariates are significant and explain 80% of the variation. The value of Q4 is smaller in females than it is in males, decreases when age increases, and is smaller in smokers. For the binary trait D, Age and Smoking are significant. The probability of the event {D = 1} increases when age increases and is higher in smokers.

### Comparison of SNP-based and gene-based association studies

We first check the effect of true associated SNPs using a linear model. The results show that 35% of the variation in Q1 can be explained by the 39 true associated SNPs in 9 genes; 22% of the variation in Q2 can be explained by the 72 true associated SNPs in 13 genes; there are no true associated SNPs for Q4. The variation in D explained by true associated genes cannot be calculated because the original values of the liability used to determine D are unknown.

Table 1 lists the number of positive genes for each method applied to each trait as well as the observed FDR. From this table, we see that the observed FDRs can be much larger than the values to be controlled. For example, in Q1 when *α* = 0.25 and the method is SNP-based, the observed FDR is 0.849, although in theory we should have FDR <*α* if all genes are independent. There are two possible reasons for this result. First, the total number of tests is only 86, which is not large compared to the number of associated genes. Second, these tests are not independent. Although the BH procedure to control FDR is not satisfied, we find that in the gene-based studies the situation can be better than in the SNP-based one. The reason may be that independence between genes is easier to satisfy than independence between SNPs. Several investigators have discussed controlling the FDR in correlated tests [8-11], but we do not discuss the issue here because of space limitations.

**Table 1:** Number of positive genes and estimated FDR

Trait	Method	*α* = 0.25		*α* = 0.5		*α* = 0.75	
	
		No. Pos	FDR	No. Pos	FDR	No. Pos	FDR
Q1	SNP	35.185	0.849	51.625	0.874	65.875	0.884
	GL	31.790	0.791	47.935	0.837	65.100	0.869
	G2	26.785	0.777	39.730	0.823	47.255	0.841

Q2	SNP	3.975	0.281	11.060	0.486	29.905	0.655
	GL	4.175	0.297	12.350	0.503	29.315	0.662
	G2	4.370	0.351	13.755	0.545	27.760	0.684

Q4	SNP	0.325	–	1.215	–	3.210	–
	GL	0.365	–	1.120	–	3.370	–
	G2	0.415	–	1.325	–	2.900	–

D	SNP	3.695	0.324	12.690	0.519	33.940	0.580
	GL	5.225	0.261	17.355	0.374	37.050	0.476
	GS	4.945	0.274	13.615	0.377	34.330	0.437
	G2	5.575	0.284	19.160	0.416	33.880	0.467

SNP, GL, G2, and GS are the SNP-based, gene-based linear, gene-based two-step, and gene-based similarity methods, respectively. *α* is the predefined value in the BH procedure. “No. POS” is the average number of positives over 200 replicates; and the FDR is the observed false discovery rate.

**Figure 1:**
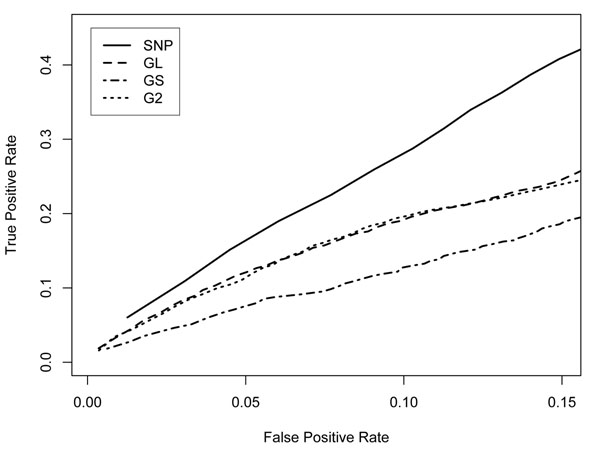
**ROC plot for trait D** The *x*-axis is the average observed false positive rate over 200 replicates and 50 nonassociated genes. The *y*-axis is the average observed true positive rate (or power) over 200 replicates and 36 truly associated genes. SNP, SNP-based method; GL, gene-based linear method; GS, gene-based similarity method; G2, gene-based two-step method.

We made a power comparison for traits Q1, Q2, and D. The results are similar. We show results only for trait D in the following discussion for conciseness. Figure 1 compares the power of the SNP- and gene-based methods for the binary trait D. It is obvious that the SNP-based method is most powerful on average for an association test. The GL and G2 methods are similar and are better than the GS method. However, no method is superior in all the situations. Table 2 gives the power comparison for each true associated gene. We list only the genes with power greater than or equal to 0.3 when the observed FPR is 0.1. From this table, we see that the power to detect true associated genes for trait D is generally low. For the genes for which an associated SNP is not rare, such as *FLT1* (SNP C13S523, MAF = 0.067), the SNP-based method is preferred because it is simpler and more powerful. However, some genes, such as *FLT4* and *HIF1A*, have multiple rare alleles (MAF ≤ 0.012) that contribute to the trait; in this case the gene-based methods can have more power than the SNP-based method. Therefore we conclude that the gene-based association might be able to pick up some association signals that are neglected in a SNP-based search.

**Table 2:** Power to identify each associated gene for trait D

Gene	Observed FPR = 0.05				Observed FPR = 0.1			
	
	SNP	GL	GS	G2	SNP	GL	GS	G2
*BCHE*	0.155	0.12	0.005	**0.195**	**0.375**	0.25	0.04	0.285
*FLT1*	**0.9**	0.705	0.83	0.335	**0.965**	0.785	0.915	0.505
*FLT4*	0.14	0.17	0.005	**0.2**	0.2	0.26	0.01	**0.37**
*HIF1A*	0.1	0.13	0.015	**0.16**	0.185	0.22	0.03	**0.335**
*KDR*	0.2	**0.42**	0.06	0.41	**0.645**	0.585	0.12	0.585
*LPL*	**0.255**	0.04	0.075	0.055	**0.4**	0.095	0.145	0.135
*HSP90AA1*	**0.11**	0.035	0.025	0.06	**0.305**	0.075	0.09	0.075
*PIK3C2B*	**0.37**	0.085	0.095	0.105	**0.665**	0.2	0.21	0.175
*PIK3C3*	**0.475**	0.44	0.08	0.4	0.565	**0.585**	0.14	0.58
*PRKCA*	0.125	0.225	**0.255**	0.165	0.3	0.36	**0.41**	0.31
*PTK2B*	0.4	0.255	0.035	**0.44**	**0.61**	0.41	0.07	0.595
*SOS2*	0.24	0.09	**0.29**	0.145	0.375	0.205	**0.415**	0.3
*VEGFC*	0	**0.275**	0	0.26	0.05	**0.385**	0	0.335
*VLDLR*	**0.19**	0.05	0.02	0.065	**0.32**	0.085	0.06	0.115
*VNN1*	**0.26**	0.175	0.045	0.065	**0.37**	0.25	0.095	0.13

SNP, GL, GS, and G2 are the SNP-based, gene-based linear, gene-based similarity, and gene-based two-step methods, respectively. The boldface values are the maximum power of these four methods.

### Genome-wide search for associated genes

TableÂ 3 lists the results from the genome-wide association studies based on SNPs and genes for trait Q1 (9 associated genes out of 3,205). The control of FDR using the BH procedure almost completely fails here. For example, when we use *α* = 10^−5^, the observed FDRs are 2/3, 1/3, and 3/4 for SNP, GL and G2 methods respectively. Because it is extremely hard to find associated genes here, we might instead lower our goal and try to get a (fairly large) subset of genes containing associated ones. For example, when *α* = 0.5, there are 6 (out of 1,272), 7 (out of 1,253), and 8 (out of 977) true associated genes using the SNP-based, GL and G2 methods respectively. It seems that in this data set the G2 method is the best, the GL method is the second best, and the SNP-based method is the worst, because the G2 method reports fewer significant genes, which contain more true associated ones instead.

**Table 3:** Comparison of number of significant genes using trait Q1

*α*	SNP			GL			G2		
	
	True	False	Total	True	False	Total	True	False	Total
1 × 10^−5^	1	2	3	2	1	3	1	3	4
1 × 10^−4^	1	4	5	2	6	8	1	4	5
1 × 10^−3^	2	19	21	2	23	25	1	12	13
0.01	2	73	75	3	101	104	1	75	76
0.10	4	389	393	5	397	402	4	303	307
0.25	5	718	723	6	695	701	7	626	633
0.50	6	1,266	1272	7	1,246	1,253	8	969	977

SNP, GL, and G2 are the SNP-based, gene-based linear, and gene-based two-step methods, respectively. The “True” columns are number of true associated genes; the “False” columns are number of false-positive genes; the “Total” columns are the total number of positive genes detected.

The problems of weak power and a high FPR are much more severe for the other traits when the genetic variation is lower. For example, for the binary trait D, we applied all four methods to data set 1. The results from these methods are similar. For the GS method, in the 100 most significant genes there are 3 true associated ones, and in the 1,000 most significant genes there are 14 true associated ones. Note that if we equally likely select 1,000 from 3,205 genes (37 true associations), the expected number of true associated genes reported is 11.5, which is less but not very much less than 14. This indicates that *p*-values here are not very informative. Therefore it is almost impossible to identify true associated genes in this situation.

## Conclusions

The detection of an association signal for rare variants is a challenging problem because of insufficient data. The SNP-based association study is more powerful than gene-based methods on average. However, when multiple rare variants contribute to a trait, the gene-based association study not only gives more reliable control over FDR but also is possible to pick up association signals neglected by a SNP-based study.

## Competing interests

The authors declare that there are no competing interests.

## Authors’ contributions

LT performed the statistical analysis and drafted the manuscript. JY participated in the group discussion and helped with the statistical analysis and manuscript. BT requested data and helped to draft the manuscript. RSC helped to draft the manuscript. All authors read and approved the final manuscript.
